# Single-cell transcriptomic atlas uncovers immune/structural lineage remodeling and fibrosis in *Klebsiella pneumoniae-*infected porcine lungs

**DOI:** 10.1080/21505594.2026.2747487

**Published:** 2026-10-12

**Authors:** Wei Wang, Junhui Zhu, Sibo Zhu, Changyou Xia, Xuan Jiang, Chuntong Bao, Ziheng Li, Rining Zhu, Tong Wu, Nan Zhang, Qila Sa, Shaopeng Wei, Brendon Scicluna, Fengyang Li, Liancheng Lei, Na Li

**Affiliations:** a State Key Laboratory for Diagnosis and Treatment of Severe Zoonotic Infectious Diseases, Key Laboratory for Zoonosis Research of the Ministry of Education, Institute of Zoonosis, and College of Veterinary Medicine, Jilin University, Changchun, China; b MOE Key Laboratory of Contemporary Anthropology, College of Life Sciences, Fudan University, Shanghai, China; c State Key Laboratory for Animal Disease Control and Prevention, National Poultry Laboratory Animal Resource Center, Heilongjiang Provincial Key Laboratory of Laboratory Animal and Comparative Medicine, Harbin Veterinary Research Institute, Chinese Academy of Agricultural Sciences, Harbin, China; d Department of Applied Biomedical Science, Faculty of Health Sciences, Mater Dei Hospital, University of Malta, Msida, Malta

**Keywords:** Klebsiella pneumoniae, porcine lung tissue, immune response, single-cell RNA sequencing

## Abstract

*Klebsiella pneumoniae* (KP) is a predominant pathogen in respiratory infections, whose widespread antibiotic resistance poses severe public health risks and economic burdens. As the primary target of KP infection, the lung contains diverse immune cells, while pulmonary cellular responses to KP remain poorly understood. This study integrated single-cell RNA sequencing, flow cytometry, RT-qPCR, and immunofluorescence to investigate porcine lung cell response from healthy pigs and those infected with multidrug-resistant, highly virulent KP. Post-infection, myeloid cells (neutrophils, monocytes, and interstitial macrophages) were rapidly activated with upregulated signatures related to neutrophil migration and interferon production. Alveolar macrophages (AM) exhibited marked heterogeneity: KP infection was associated with apoptosis in proliferating, monocyte-derived, and tissue-resident AM subsets, indicating a disruption of homeostasis, while infection-specific subsets (AM_MHCII, AM_CXCL2) displayed enhanced antigen presentation, inflammatory activity, and pro-fibrotic potential. Under homeostasis, γδ T and CD8αα^+^ T cells were the predominant T cell populations. After infection, CX3CR1^−^CD8αα^+^ T cells were activated, and CD8^+^ γδ T cells increased their interactions with myeloid cells, predominantly through ligand-receptor pairs such as TNF-TNFRSF1B and IFNG-IFNGR1/2. Furthermore, KP induced lung fibroblast proliferation and activation via the ROS-mediated TGF-β/SMAD pathway, WNT pathway, and aerobic glycolysis. Concurrently, infection reduced the proportion of ATII and transitional AT0 cells, which may impair their differentiation into ATI cells and compromise epithelial barrier function. Thus, this study is the first to characterize the dynamics of KP infection in porcine lungs at single-cell resolution. It advances our understanding of KP-host interactions and provides a framework for anti-KP therapeutic development.

## Graphical abstract

**Figure:**
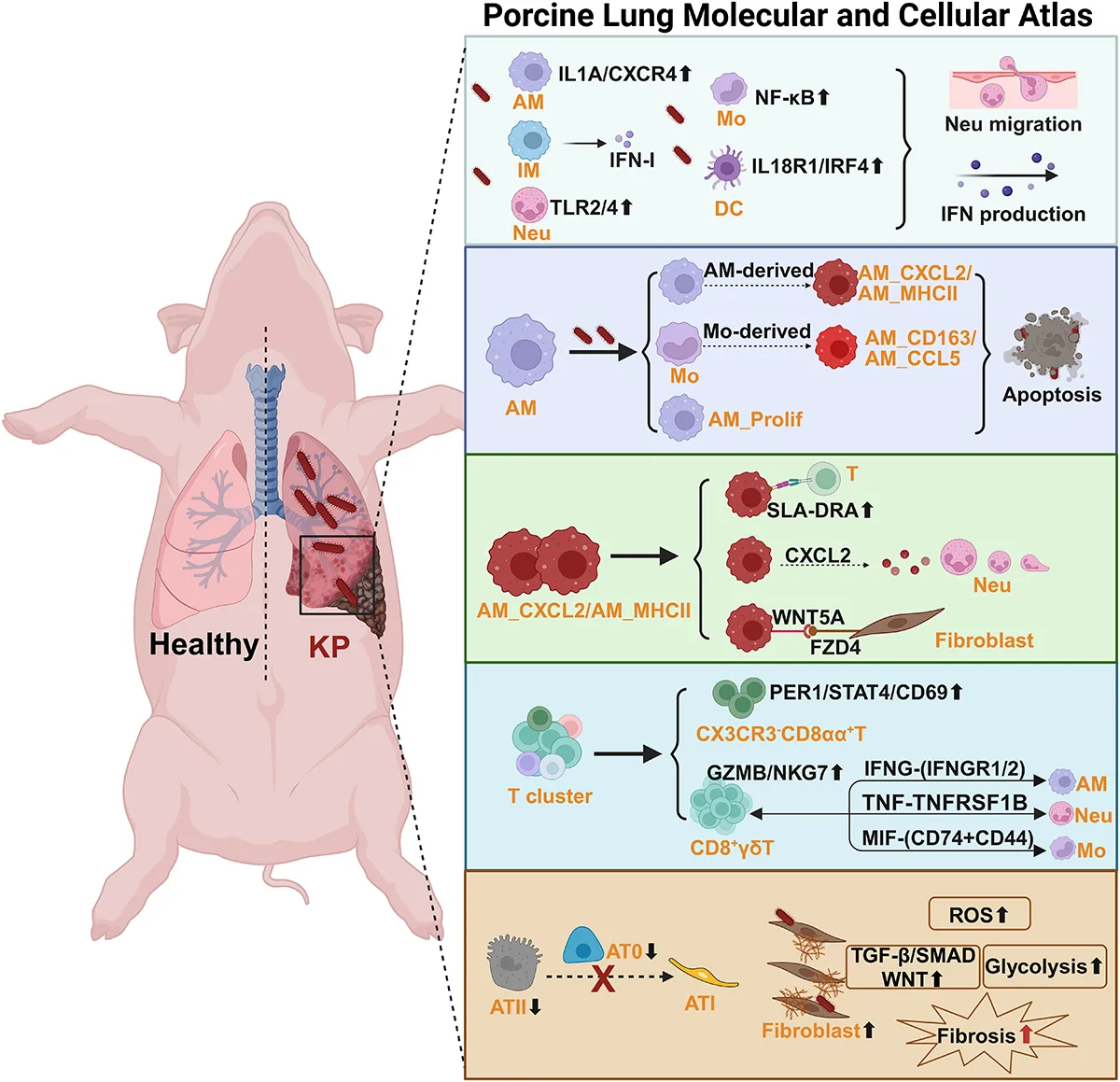

## Introduction

*Klebsiella pneumoniae* (KP), a Gram-negative opportunistic pathogen, is a leading cause of hospital-acquired pneumonia globally and community-acquired pneumonia in Asia [1]. Despite antibiotic treatment, the mortality rates of KP-induced pulmonary infections are as high as 50%. Meanwhile, KP is a major determinant of bacterial pneumonia secondary to COVID-19 [2]. Beyond its impact on humans, KP poses a significant threat to livestock health, causing bovine mastitis [3], ovine pneumonia [4], porcine sepsis or pneumonia [5,6], with particularly high subclinical infection rates in swine. The subclinical infection predisposes pigs to severe disease under immune impairment [6,7]. Critically, the global spread of hypervirulent and multidrug-resistant KP strains, including zoonotic transmission of human-derived lineages, underscores its escalating public health risk [6,8].

Successful KP colonization hinges on dynamic interactions with host immune cells, including macrophages, neutrophils (Neu), and monocytes (Mo). However, most research focuses on single-cell types rather than systemic immune dynamics using mouse models. Alveolar macrophages (AM) exert the roles in phagocytic killing and immune modulation to limit tissue damage, whereas interstitial macrophages (IM) may paradoxically facilitate infection [9]. Neu and Mo are rapidly recruited for bacterial clearance via phagocytosis [10], and γδ (but not αβ) T cells confer protection during KP pneumonia [11]. Despite this, systematic analysis of the pulmonary cellular response to KP, particularly within the lung’s unique immune architecture, remains insufficient.

The scarcity of human KP-infected lung tissue has limited progress in translational research. While murine models remain the dominant tool for KP studies, they exhibit significant anatomical and immune disparities compared to humans, hindering insights into large mammals [12]. In contrast, pigs share key similarities with humans in lung structure (including lobar/bronchial anatomy), organ size, and immune function, making them a superior model for human-relevant KP research [13]. Notably, recent KP isolations from swine farms across China, Thailand, and the UK confirm pigs as susceptible hosts [7,14–16], further highlighting their value for characterizing pulmonary immunity and pathogenesis. However, the model of pigs remains underutilized in the context of KP infection. Here, we employed single-cell RNA sequencing to conduct a comprehensive landscape analysis of pulmonary cellular responses in healthy piglets and those infected with human-derived, hypervirulent, and multidrug-resistant KP. Meanwhile, we revealed KP infection-induced cell response characteristics and cellular crosstalk among immune and structural cells in porcine lungs as a large mammalian model.

## Materials and methods

### Origin and identification of bacterial strains

The KP strain employed in the current study was obtained from clinical sputum specimens collected at Bethune First Hospital of Jilin University. Based on *WZI* gene sequencing, PCR amplification of virulence genes, and the string test, the KP strain used in this study was confirmed to be a hypervirulent K2 serotype with O1 O-antigen (Figure S1A-C; Tables S1 and 2).

### Antibiotic susceptibility testing

The agar disk diffusion method was employed to determine the antibiotic susceptibility of KP. 150 μL of bacterial culture (12 h incubation) was evenly spread on plates, and after air-drying, 24 antibiotic disks were uniformly placed. Plates were incubated inverted at 37°C for 24 h, then inhibition zone diameters were measured, with strain resistance determined according to NCCLS standards. Antimicrobial susceptibility testing was performed using commercial discs with standard concentrations as recommended by the manufacturer. The strain used in the current study was multidrug-resistant (triplicate experiments, Table S3).

### Establishment of infection model

Six 45-day-old male Rongchang pigs, provided by the Harbin Veterinary Research Institute, were randomly allocated into two groups (KP-infected group, *n* = 3; Control group, *n* = 3). Enzyme-linked immunosorbent assay (ELISA) was performed to detect KP in all experimental animal samples using laboratory-prepared specific antigen-coated plates, and the results confirmed that all samples were KP-negative. For infection modeling, piglets in the KP group were intranasally inoculated with 1 mL of K2 serotype bacterial suspension at a concentration of 2 × 10^7^ CFU per pig, while those in the Control group received an equal volume of phosphate-buffered saline (PBS). At 96 h post-inoculation, pigs were euthanized with a sodium pentobarbital solution (sodium pentobarbital solution), followed by immediate collection of lung tissue for single-cell suspension preparation. Clinical scores were evaluated according to the method described by Sibila et al. [17], and continuous clinical monitoring was initiated immediately after infection (Figure S1D). The scoring criteria were as follows: score 0: no signs of disease; score 1: increased breathing rate, occasional coughing and mild depression; score 2: abdominal breathing, usually lying down, standing when gentle stimulated; score 3: regular coughing or holding up on forelegs in a sitting position or markedly depressed or reluctant to stand up and with an increased heart rate (>110 beats per min); score 4: clinical score of 3 plus deteriorating toward showing signs of severe dyspnea.

### Preparation of single-cell suspensions for scRNA-seq

Lung tissues (focusing on removing 1 cm from the primary area of the lesions caused by KP) were washed three times with pre-cooled PBS, cut into 1 mm pieces with scissors, and placed in preheated culture medium containing 25 U/mL DNase I (Solarbio, China), 300 U/mL collagen type VIII (Sigma, USA) for enzymatic digestion (37°C, 20 min). The suspension was further mechanically dissociated, filtered through a 70 μM strainer, and neutralized with 1x PBS containing 10% fetal bovine serum (FBS). The cell suspension was enriched by centrifugation at 350 g for 7 min at 4°C. Erythrocyte lysis was performed on the cell suspension (Solarbio, China). After removing the dead cells using OptiPrep medium (Axis-Shield, Scotland), the cells were counted and immediately analyzed by scRNA-seq. Chromium Single Cell 3ʹ Library Kit, Gel Beads, Multiplex Kit, and Chip Kit (10X Genomics) were used for single-cell 3ʹ library construction, with all operations conducted per the manufacturer’s instructions. The library pool was sequenced using an Illumina NovaSeq 6,000 instrument with 150-base-pair paired-end reads. Subsequently, each sample of the raw sequence data was mapped to the pig reference genome Sscrofa11.1.104 with Cell Ranger (v.6.1.2). The gene count expression matrices were constructed by the CellRanger count function.

### scRNA-seq data processing

Downstream analysis was performed using the “Seurat” package (v.5.0.1) in R (v.4.2.3) to create and analyze Seurat objects [18]. Quality control removed genes detected in ≤3 cells and cells expressing ≤ 200 genes. Cells containing 200–4,000 genes were retained, and unique molecular identifiers (UMIs) and mitochondrial genes were less than 30% for further analyses. Doublet identification and removal were performed using DoubletFinder (version 2.0.3) to ensure data quality for subsequent analyses (pN = 0.25; pK = 0.2). After removing low-quality cells, expression was homogenized using the LogNormalize method of the Seurat software “Normalization” function (scale factor = 10,000). The VST method in the FindVariableFeatures function was used to select 2,000 genes. Linear dimensionality reduction was performed on scaled gene expression matrices using the RunPCA function. Subsequently, the FindNeighbors and FindClusters (resolution = 0.2) functions were applied for cluster identification. The RunUMAP function was executed for a two-dimensional visualization of cell clusters. Inter-sample relationships were assessed by calculating Spearman correlation coefficients of the top 5,000 highly variable genes, and heatmaps of sample correlations were generated using the R heatmap package. More details about the Seurat analyses can be found on the website tutorial (https://satijalab.org/seurat/articles/pbmc3k_tutorial.html).

### Classification and identification of cell populations and subpopulations

The FindAllMarkers function was used to identify differentially expressed genes (DEGs) between cell populations, followed by manual annotation based on canonical marker genes. Selected subpopulations (e.g. AM, T, Club, and ATII cells) were extracted for re-clustering and dimensionality reduction. Each subpopulation was re-analyzed using ScaleData, RunPCA, RunUMAP, FindClusters, and FindAllMarkers, followed by manual subpopulation annotation based on marker genes (Table S4). The FindMarkers method uses the Wilcoxon rank-sum test as default.

### GO and KEGG pathway enrichment analysis of DEGs

The top 100 up- and down-regulated DEGs (Table S5) were subjected to pathway enrichment analysis using ClusterProfiler (v4.6.2) and Org.Ss.eg.db (v3.16.0), and results were visualized using ggplot2 (v3.4.4).

### Defining cell state scores

We employed cellular scoring to evaluate the expression level of predefined gene sets in individual cells [19]. The R package AUCell (v1.26.0) was used to calculate cell scores for: positive regulation of innate immune response (GO:0045089), neutrophil migration (GO:1902622), type I interferon production (GO:0032606), and macrophage apoptotic process (GO:0071888).

### Trajectory analysis

To construct trajectories, Mo and AM cell subpopulations, along with Club and ATII cells, were extracted. Monocle3 (v1.3.1) was employed to create CellDataSet objects, perform dimensionality reduction, and generate cellular trajectories. Based on established biological knowledge [20,21], the root node of the trajectory was manually selected to construct the cellular trajectory, followed by identification of pseudotime-dependent differentially expressed genes and the visualization of gene expression trends.

### Ligand-receptor-based cell-cell interaction analysis

Using CellChat (v1.6.1), expression matrices and cell annotations from Seurat objects were extracted to construct Cellchat objects, with subsequent incorporation of human-curated ligand-receptor pairs (the built-in database of CellChat only supports human, mouse, and zebrafish, with no official annotation database for pig available) for intercellular communication network inference, and visualization of receptor-ligand interactions through bubble plots.

### Hematoxylin and eosin staining, along with Sirius red staining

Briefly, porcine lung tissues were dehydrated in ethanol, paraffin-embedded, and sectioned at 5 μM thickness. Sirius red staining was performed using a commercial kit (Solarbio, S8060-5). For H&E staining, sections were dewaxed in xylene and rehydrated through an ethanol series, followed by hematoxylin staining for 3–5 min, rinsed in tap water, differentiated in 1% acid alcohol for 5 s, and counterstained with eosin for 2 min before water washing. Finally, stained sections were dehydrated through absolute ethanol I, II, III, and xylene I, II (5 min each). After mounting, images were captured using an Olympus microscope (Olympus, Japan) [22].

### Apoptosis detection in porcine alveolar macrophages by flow cytometry

Porcine alveolar macrophage (PAM) cells were counted and adjusted to 1 × 10^5^ cells/well, then infected with KP at a multiplicity of infection (MOI) of 30. Apoptosis was analyzed using PE Annexin V Apoptosis Detection Kit (BD Biosciences, Franklin Lakes, NJ, USA) on a flow cytometer (Beckman, USA) following the manufacturer’s protocol. Briefly, at 2 h and 4 h post-infection, cells were trypsinized (without EDTA) and suspended in Annexin V binding buffer, and stained with Annexin V-PE and propidium iodide (PI) at room temperature for 15 min. Samples were diluted with 400 μL binding buffer per tube before acquisition on the flow cytometer. The data were analyzed with FlowJo v10.6.2 (FlowJo software, BD Biosciences).

### Isolation, culture, and infection of porcine pulmonary fibroblasts

Briefly, porcine lungs were excised and washed three times with PBS containing antibiotics (Penicillin-Streptomycin-AmpHotericin B Solution, Beyotime). The tissues were then minced into small pieces and digested in medium containing 0.01 mg/mL collagenase IV (Solarbio) at 37°C for 20 min. Digestion was terminated by adding an equal volume of culture medium containing 10% fetal bovine serum (FBS). Subsequently, the cell suspension was filtered through a 100 μM cell strainer, centrifuged at 500 × g for 8 min at 4°C, and washed three times. The cells were then seeded into 6-well culture dishes at a density of 1.5 × 10^5^ cells per well. Fibroblasts were obtained after 3–5 passages. N-acetylcysteine (NAC, MCE, HY-B0215) was dissolved in RNase-free water, and PFK-15 (MCE, HY-12204) was dissolved in dimethyl sulfoxide (DMSO). The concentrations used were 0.5 mM and 0.25 mM for NAC, and 0.5 μM for PFK-15. In the experiment, cells were pre-treated with both reagents for 1 h, and the same concentrations of these reagents were also added during the KP infection. Primary fibroblasts were cultured to 85% confluency in 24-well plates (1 × 10^5^ cells/well) and subsequently infected with KP at an MOI of 30. After infection, cellular samples were harvested at precisely timed intervals (2, 4, and 6 h) for subsequent downstream analysis.

### Measurement of ROS production in fibroblasts

ROS levels in KP-infected fibroblasts were measured using ROS Assay Kit (Beyotime, S0033S-1). Following collection, cells were resuspended in diluted DCFH-DA fluorescent dye and incubated (37°C, 20 min) with intermittent mixing (every 3–5 min) to ensure uniform probe distribution. After three PBS washes, fluorescence was detected by flow cytometry at 488/525 nm.

### Microplate-based assay for L-lactate dehydrogenase (L-LDH) and pyruvate kinase (PK) activity measurement

Fibroblast lysates were centrifuged to obtain supernatants for L-LDH (Boxbio, AKCO003M) and PK (Boxbio, AKSU066M) activity assays following the manufacturer’s protocols.

### RNA extraction and RT-qPCR

Fibroblasts were collected and lysed in RNAiso Plus reagent (Takara, 9108) for RNA extraction. Briefly, 1 mL RNAiso Plus was added to the cell pellets in microtubes, vortexed, and incubated at room temperature for 5 min. 200 μL chloroform was added per tube, vortex-mixed, incubated at room temperature for 10 min, then centrifuged at 12,000 g for 15 min at 4°C. The aqueous phase was transferred to new tubes, mixed with an equal volume of isopropanol, stored at −20°C for 20 min, then centrifuged at 12,000 g for 15 min at 4°C. Finally, RNA pellets were washed with 75% ethanol and dissolved in RNase-free water.

RNA was reverse transcribed into cDNA using the PrimeScript RT reagent kit (TaKaRa, RR037A). cDNA, primers, and SYBR Green Premix were mixed in 10 μL reaction volumes and amplified using the QuantStudio 1 qPCR system. Relative gene expression was calculated using the 2−ΔΔCt method, with primer sequences listed in Table S6.

### Immunohistochemistry

Before antigen retrieval, paraffin sections were deparaffinized and rehydrated. OCT-embedded sections were thawed and washed with PBS. Antigen retrieval was performed in 10 mM sodium citrate (pH 6.0) or Tris-EDTA (pH 9.0) buffer at 95°C for 15 min. Sections were washed with PBS, permeabilized, and blocked with 1% BSA / 0.1% Triton X-100 in PBS at room temperature for 30 min. Primary antibodies, including lab-prepared rat anti-SCGB3A2 (1:100), rabbit anti-SFTPC (1:100), commercial rabbit anti-CD68 (Proteintech, 1:2,000), and mouse anti-CD3 (Bio-Rad, 1:100), were applied at 4°C overnight. After three washes with PBS containing 0.1% Triton X-100 (PBST), fluorescent secondary antibodies-Alexa Fluor 647/APC-conjugated donkey anti-rabbit IgG (BioLegend, 1:100) and PE-conjugated goat anti-rat IgG (BioLegend, 1:100), were incubated for 1 h at room temperature. Following three PBST washes, specimens were coverslipped using Fluor-G mounting medium with DAPI nuclear counterstain. Digital whole-slide imaging was performed using an Olympus slide scanning system, followed by quantitative analysis.

### Statistical analysis methods

All statistical analyses were performed using R (v4.5.1) and GraphPad Prism (version 8.0.2). All experiments included three independent biological replicates (*n* = 3). For single-cell RNA-seq data, differential gene expression analysis was performed with the Wilcoxon rank-sum test using the FindMarkers function in Seurat (v5.4.0). The resulting *p*-values were adjusted for multiple comparisons using the Benjamini-Hochberg false discovery rate (FDR) correction. Differentially expressed genes were defined as those with an absolute average log_2_ fold change > 0.5 and an adjusted *p*-value (FDR) <0.05. Gene set activity scores (e.g. AUC scores) were compared between groups using the Wilcoxon rank-sum test, with *p*-values similarly adjusted for multiple comparisons across cell types or gene sets. Other inter-group comparisons are described in the respective figure legends. Throughout the study, a threshold of adjusted *p*-value (FDR) <0.05 was used to define statistical significance, unless otherwise specified.

## Results

### Overview of piglet lung cell types before and after KP infection

To elucidate pulmonary cellular responses post-KP infection, we established a KP-challenged piglet model (Figure 1(A)). Piglets in the control group exhibited no signs of disease, whereas KP-infected piglets displayed clinical symptoms (fever, tachypnea) and gross pathological lesions (pulmonary petechiae), with KP abundantly detected in BALF and lung homogenates (Figure 1(B–D)) and Supplemental Figure 1(C)). H&E staining revealed alveolar collapse, inflammatory cell infiltration, and fibrinous exudation, confirming the successful establishment of a KP pneumonia model (Figure 1(E)). We then performed scRNA-seq and data analysis on pig lung tissues at 96 h post-infection (Figure 1(A)). Inter-sample reproducibility within each experimental group (*n* = 3 biological replicates) was validated through pseudo-bulk correlation analysis, revealing strong intra-group concordance (Figure S1E). After rigorous QC filtering, 63,064 high-quality cells (40,671 from controls; 22,393 from infected piglets) were retained (Figure S1F-G). These cells were clustered into 15 distinct cell populations based on UMAP-based clustering and canonical marker analysis, including T cells (*CD3E*^*+*^ *CD3G*^*+*^, *n* = 16,111), AM (*MRC1*^*+*^ *S100A8*^*+*^, *n* = 25,044) [23,24], IM (*MARCO*^*+*^ *C1QB*^*+*^, *n* = 4,800) [24], Mo (*CD14*^*+*^ *LYZ*^*+*^, *n* = 3,426) [25], B cells (*CD79B*^*+*^ *MS4A1*^*+*^, *n* = 3,402), Plasma cells (*JCHAIN*^*+*^ *MZB1*^*+*^, *n* = 2,156), Neu (*G0S2*^*+*^ *CSF3R*^*+*^, *n* = 1,452) [26], DCs (*PLD4*^*+*^ *IRF8*^*+*^, *n* = 1,275) [27], Proliferating T cells (*STMN1*^*+*^ *TOP2A*^*+*^ *CD3E*^*+*^ *CD3G*^*+*^, *n* = 1,242), Lymphatic endothelial cells (*LYVE1*^*+*^ *MMRN1*^*+*^, *n* = 1,219) [24], ATII (*SFTPC*^*+*^ *SFTPA1*^*+*^, *n* = 827), Fibroblasts (*COL1A2*^*+*^ *COL3A1*^*+*^, *n* = 701), Vascular endothelia cells (*VWF*^*+*^ *PECAM1*^*+*^, *n* = 675), Clubs (*SCGB3A2*^*+*^ *SCGB1A1*^*+*^, *n* = 464), Ciliated cells (*C5orf49*^*+*^ *DYNLRB2*^*+*^, *n* = 270) (Figure 1(F,G)), Figure S1H; Table S4).

**Figure 1.**
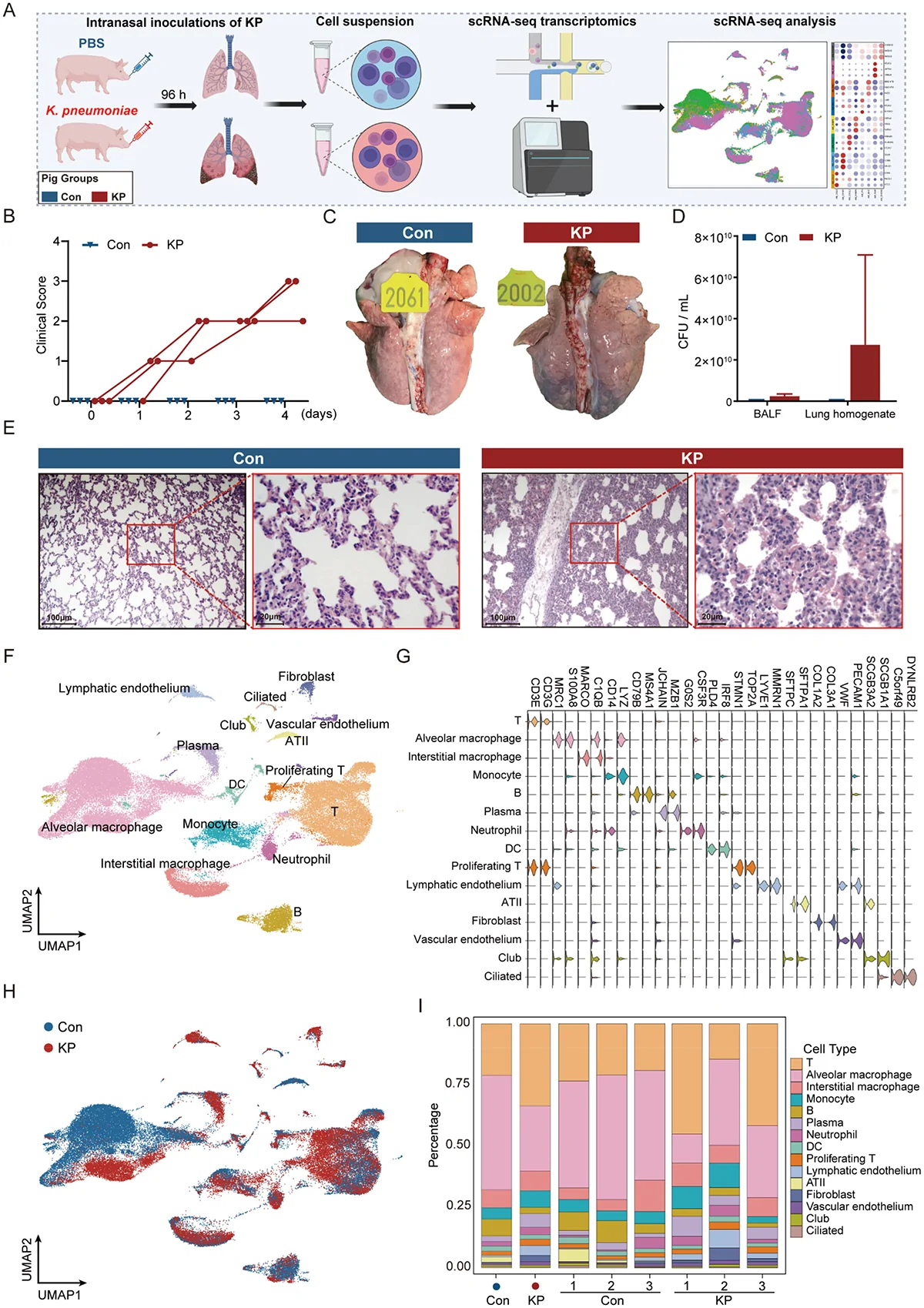
Study design and KP infection-induced alterations in pulmonary cellular composition.

(A) Schematic illustration of experimental design. A KP infection model was established in piglets, and scRNA-seq was performed to comprehensively profile lung cells in infected and control groups. Figure created with BioRender.com. (B) Line graph showing clinical score progression post-KP infection. (C) Gross pathological changes in Con and KP-infected porcine lungs. (D) Bacterial loads in BALF and lung homogenates from KP-infected piglets. Data are presented as mean ± SEM (*n* = 3 biological replicates per group). Unpaired, two-tailed Student’s t-test. (E) H&E-stained lung sections from Con and KP-infected piglets. (F) UMAP projection visualizing distinct cell clusters from control and infected groups. (G) Violin plots showing expression distributions of canonical markers across cell clusters. (H) UMAP plot showing the origins of cell clusters. (I) Proportional distribution of cell types across individual samples.

We further analyzed the changes in the proportion of these cellular subpopulations between control and infected groups (Figure 1(H,I)). Following KP infection, the proportions of IM, Mo, and Neu increased, while that of AM was markedly reduced. Among lymphocytes, the proportions of T cells and plasma cells increased, whereas B cells decreased (Figure 1(H,I)), Figure S1I). Structural cell analysis revealed expanded lymphatic endothelia, vascular endothelia, and fibroblasts, but diminished ATII, Club, and Ciliated cells post-infection (Figure 1(H,I)), Figure S1J). Notably, ATII and Ciliated cells, critical for pulmonary homeostasis, were nearly depleted post-infection, indicating profound structural and functional disruption. Collectively, these findings demonstrate that KP infection disrupts pulmonary cellular equilibrium, driving the selective expansion and remodeling of immune cell subsets.

### KP infection upregulates the function of lung myeloid cells in inducing monocyte and neutrophil migration alongside interferon production

Following KP infection, the abundance of myeloid cell subpopulations changed significantly (Figure 2(A)). To investigate their functional reprogramming, we performed GO enrichment analysis on differentially expressed genes (DEGs) from the five myeloid subsets (Table S5). AM, the lung-resident professional phagocytes, showed upregulated expression of genes encoding inflammatory mediators (e.g. *IL1A*, *NFKBIA*) and the chemokine receptor *CXCR4* post-infection. KP infection also enhanced the expression of complement activation genes (e.g. *C1QA*, *C1QB*, *FCN*) in AMs, which may facilitate phagocytosis and bacterial clearance (Figure S2A). GO analysis confirmed the enrichment of AM-upregulated genes in immune activation pathways, consistent with their immunomodulatory role (Figure 2(B)). In contrast, IM upregulated type I interferon (IFN-I)-associated genes (e.g. *ISG15*, *IRF1*, *IRF7*). Enrichment analysis identified IFN-I signaling activation as a hallmark of KP-infected IMs (Figure 2(C), Figure S2B), suggesting their potential contribution to pulmonary IFN-I production, a pathway critical for host defense. Additionally, upregulated genes in IM were enriched in pathways related to antigen processing/presentation (e.g. *TAP1*, *IFI30*, *HSPD1*) and antimicrobial defense (Figure 2(C), Figure S2B).

**Figure 2.**
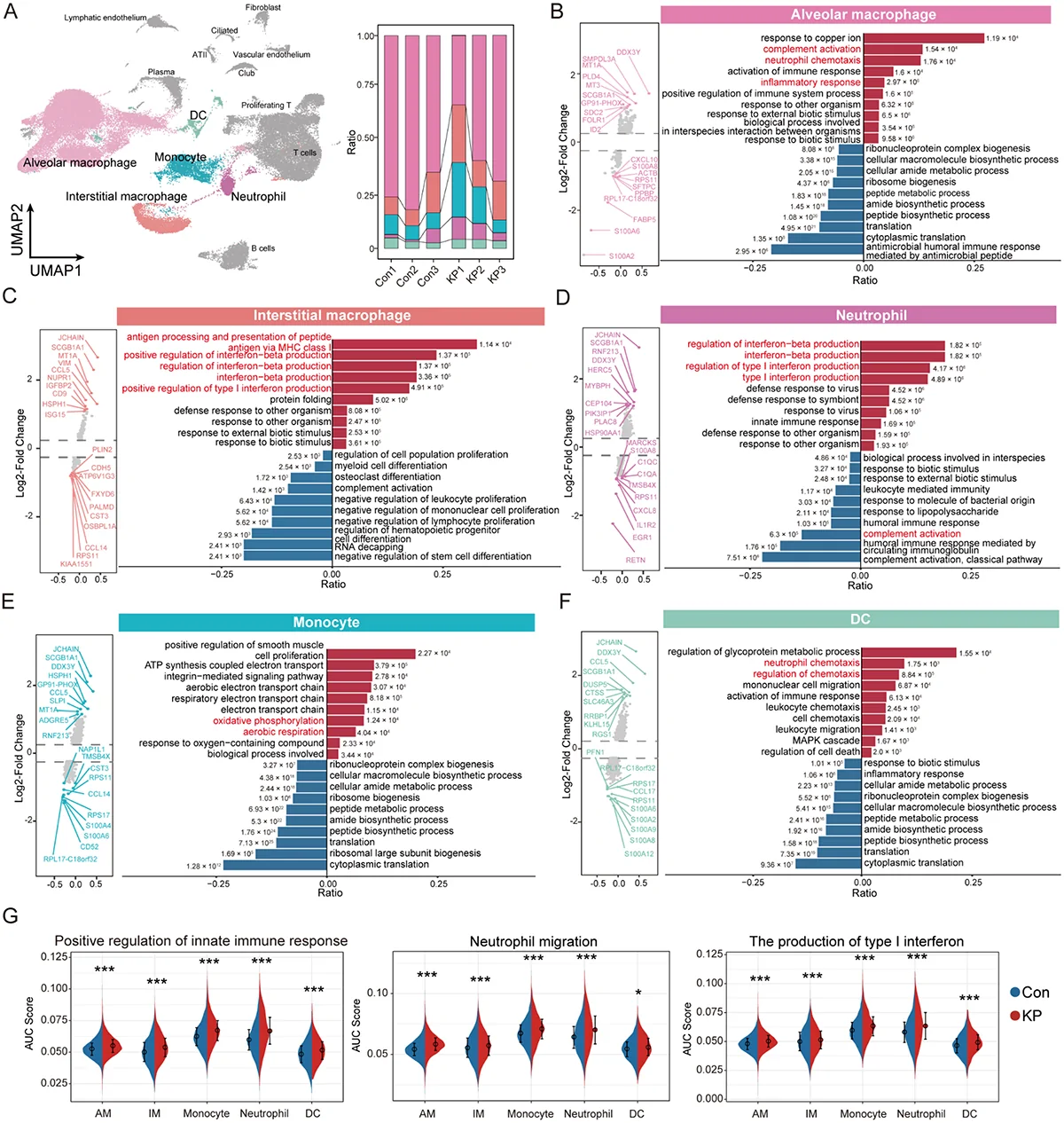
Immune response characteristics of myeloid cells induced by KP infection.

(A) UMAP visualization of myeloid cells and their abundance changes post-infection. (B-F) Differentially expressed genes (top 10) and GO enrichment results (top 10) in each myeloid subset between KP-infected and control groups. (G) Violin plots comparing gene signature activity scores for innate immune response, neutrophil migration, and type I interferon production between KP-infected and control groups across myeloid cell subsets. Data are presented as half-violin plots. Statistical significance for each cell type was determined by the Wilcoxon rank-sum test, with *p*-values adjusted for multiple comparisons across all cell types using the Benjamini-Hochberg false discovery rate (FDR) correction. ns FDR > 0.05, *FDR < 0.05, **FDR < 0.01, ***FDR < 0.001.

Beyond macrophages, the rapid recruitment of Neu and Mo is also critical for controlling KP infection. Post-infection, these Neu exhibited elevated expression of pattern recognition receptors (e.g. *TLR2*, *TLR4*), indicating activation of Toll-like receptor (TLR) signaling and inflammatory initiation (Figure S2C). Neu also upregulated genes involved in oxidative phosphorylation (e.g. *ND4*, *COX1*), which are essential for metabolic homeostasis, migration, and cytokine secretion (Figure S2C). Similar to IM, Neu-upregulated genes were enriched in IFN-I production and antimicrobial pathways. However, unlike AM, Neu downregulated complement components post-infection (Figure 2). Mo, a heterogeneous population, rapidly infiltrated the lungs during KP infection. KEGG pathway analysis revealed enrichment of NF-κB and Toll-like receptor signaling in infected Mo, suggesting a pro-inflammatory phenotype. Mo also upregulated chemokines (e.g. *CCL5*, *CCL21*) and adhesion molecules (*ITGA4*), which are associated with T cell recruitment and activation (Figure 2(E), Figure S2D-E). Furthermore, Mo-upregulated genes were enriched in oxidative stress pathways, highlighting their role in antimicrobial defense (Figure 2(E)). DCs increased expression of *IL18R1* and *IRF4*, which may enhance T cell priming, while simultaneously upregulating genes in neutrophil chemotaxis pathways, potentially amplifying antimicrobial responses (Figure 2(F), Figure S2F). AUCell scoring confirmed the enhanced activity of innate immune response, neutrophil migration, and interferon production pathways in lung myeloid cells post-infection (Figure 2(G)). Collectively, these data indicate that myeloid cells in piglet lungs orchestrate innate immunity post-KP infection, primarily through mechanisms associated with neutrophil recruitment and interferon-mediated defenses.

### KP infection disrupts AM homeostasis and induces apoptosis through multiple pathways

AM, as the primary innate immune cells in lungs, exhibit a resting, anti-inflammatory phenotype with high phagocytic activity under physiological conditions [28]. Following KP infection, the proportion of AM significantly decreased, which was confirmed by immunohistochemistry [29] (Figure 1 and 3(A)). To investigate the mechanism underlying KP-induced AM loss, we performed subclustering analysis and identified eight functionally distinct AM subsets based on differential gene expression profiles: (1) AM_IFN (*CXCL10*^*+*^ *CXCL8*^*+*^ *IRF1*^*+*^): interferon-responsive; (2) AM_FABP5 (*FABP5*^*+*^ *PON1*^*+*^ *PTGR1*^*+*^): lipid-associated; (3) AM_CCL5 (*CCL5*^*+*^ *CXCR4*^*+*^ *RAC2*^*+*^) &; (4) AM_S100A8 (*S100A8*^*+*^ *S100A9*^*+*^ *S100A12*^*+*^): participating in immune cell recruitment and regulation; (5) AM_MHCII (*CD14*^*+*^ *SLA-DRA*^*+*^ *SLA-DMB*^*+*^): antigen-presenting; (6) AM_CXCL2 (*CXCL2*^*+*^*TNFSF13*^*+*^*NFKBIA*^*+*^): related to inflammatory responses; (7) AM_CD163(*CD163*^*+*^ *MRC1*^*+*^ *APOE*^*+*^): anti-inflammatory; (8) AM_Prolif (*STMN1*^*+*^ *PCLAF*^*+*^ *TOP2A*^*+*^): proliferative (Figure 3(B); Table S4).

**Figure 3.**
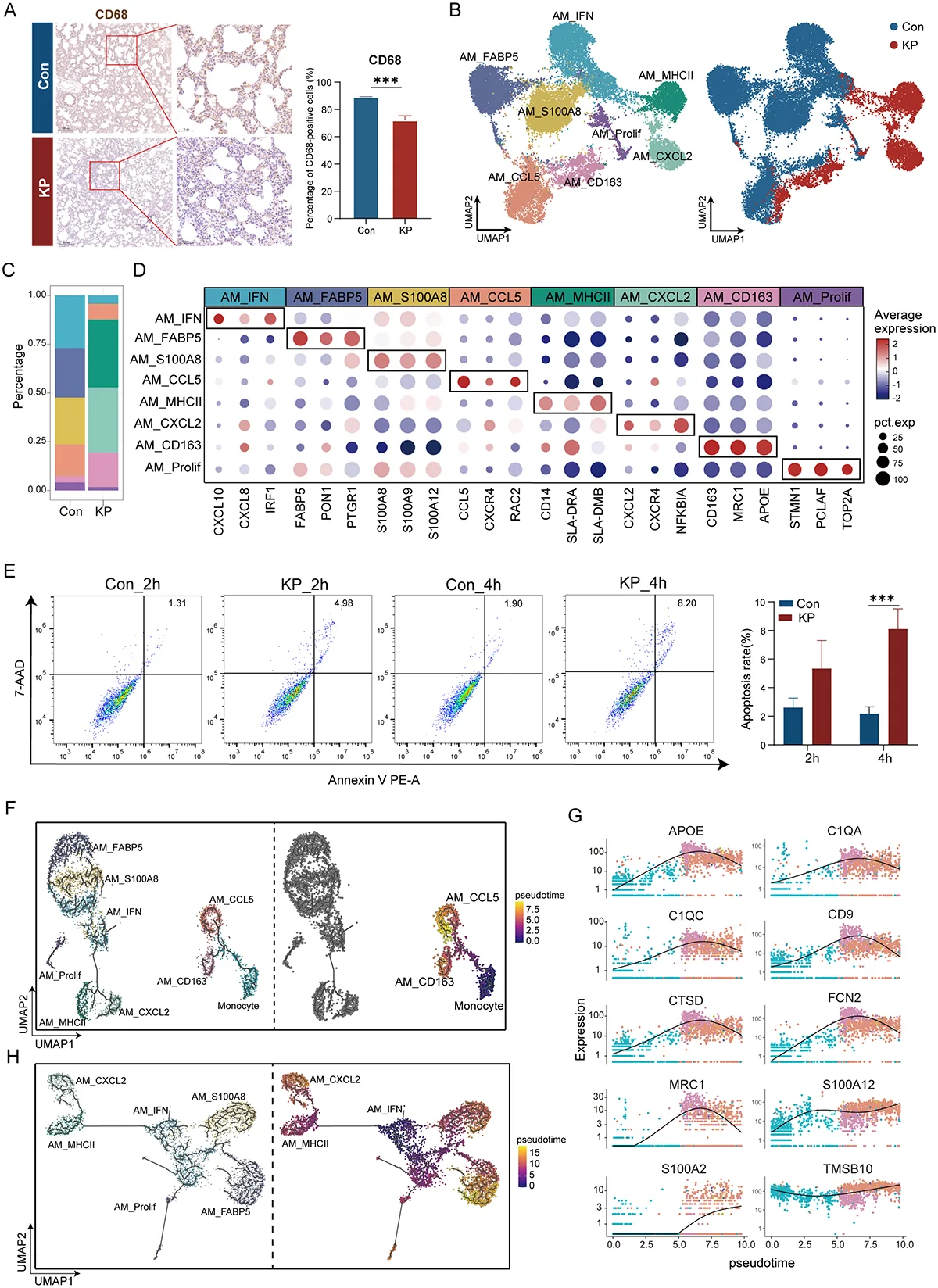
Response characteristics of alveolar macrophages in KP-infected piglet lungs.

(A) CD68 expression in lung tissues from control and KP-infected groups. For quantification, eight random visual fields per sample were selected to calculate the percentage of CD68-positive cells. ***p < 0.001. (B) UMAP projection illustrating AM subsets and their cluster identities. (C) Proportional distribution of macrophage subsets in control and KP-infected groups. (D) Bubble plot showing scaled expression levels of cell-type-specific genes in the indicated AM subsets. (E) Flow cytometric analysis of apoptosis in PAM post-KP infection. Bars show the mean ± SEM from three independent biological replicates (*n* = 3 per group). Statistical significance between Con and KP groups at each time point was assessed by the unpaired, two-tailed Wilcoxon rank-sum test. *p < 0.05, **p < 0.01. (F) Pseudotime trajectory analysis of monocytes and AM populations. (G) Expression of the top 10 dynamically regulated genes along pseudotime trajectories. (H) Pseudotime analysis of AM populations.

UMAP and cellular proportion analyses revealed a dramatic shift in the AM landscape during KP infection, characterized by a severe reduction in homeostatic tissue-resident AM and the emergence of two novel, heterogeneous subsets: AM_MHCII and AM_CXCL2 (Figure 3(B–D)). Consistent with the observed loss of AM, KEGG enrichment analysis showed significant enrichment of apoptotic pathways in AMs post-infection. This was further supported by elevated AUCell apoptosis scores in the AM_CCL5, AM_FABP5, AM_IFN, and AM_Prolif subsets (Figure S3A-B). This finding was experimentally validated *in vitro*, where KP infection induced time-dependent apoptosis in PAM (Figure 3(E)). To explore potential mechanisms of AM replenishment, pseudotime trajectory analysis reconstructed the differentiation trajectories from Mo to AM_CCL5 and AM_CD163 subsets, suggesting that circulating monocytes may contribute to repopulating the AM niche following infection (Figure 3(F)). Along these trajectories, the top differentially expressed genes included apolipoprotein (*APOE*), lipocalin-2 (*FCN2*), and complement genes (*C1QA/C*; Figure 3(G)), with APOE serving as a key checkpoint gene governing Mo-to-macrophage differentiation during pulmonary inflammation [30]. Pseudotime analysis further suggested that the two emergent AM subsets post-infection may originate from resident AM_IFN cells (Figure 3(H)). Notably, both repopulation pathways, whether Mo-derived (AM_CD163, AM_CCL5) or resident macrophage-derived (AM_MHCII, AM_CXCL2) subsets, exhibited upregulated apoptosis-related genes and apoptotic features (Figure S3C).

Taken together, these results suggest that KP infection is associated with Mo-to-AM differentiation while concurrently being linked to increased apoptosis in both resident AM and newly differentiated, monocyte-derived macrophages. The observed apoptosis in proliferating macrophage subsets further implies that KP infection may promote apoptotic cell death across multiple AM lineages.

### KP infection-specific AM subsets display enhanced antigen presentation, inflammatory responses, and pro-fibrotic tendency

To systematically characterize the potential functions of AM subsets during KP infection, we performed GO enrichment analysis of DEGs. Each subset exhibited specialized immunological roles: AM_IFN participated in immune responses by upregulating lymphocyte-mediated immunity and chemokine signaling; AM_FABP5 upregulated complement activation and humoral immunity; AM_S100A8 engaged in antimicrobial immunity via IFN-I signaling; AM_CCL5 showed enrichment in cytokine response pathways; AM_CD163 activated programmed cell death pathways; and AM_Prolif enhanced smooth muscle proliferation, Neu migration, and chemotaxis (Figure 4(A)).

**Figure 4.**
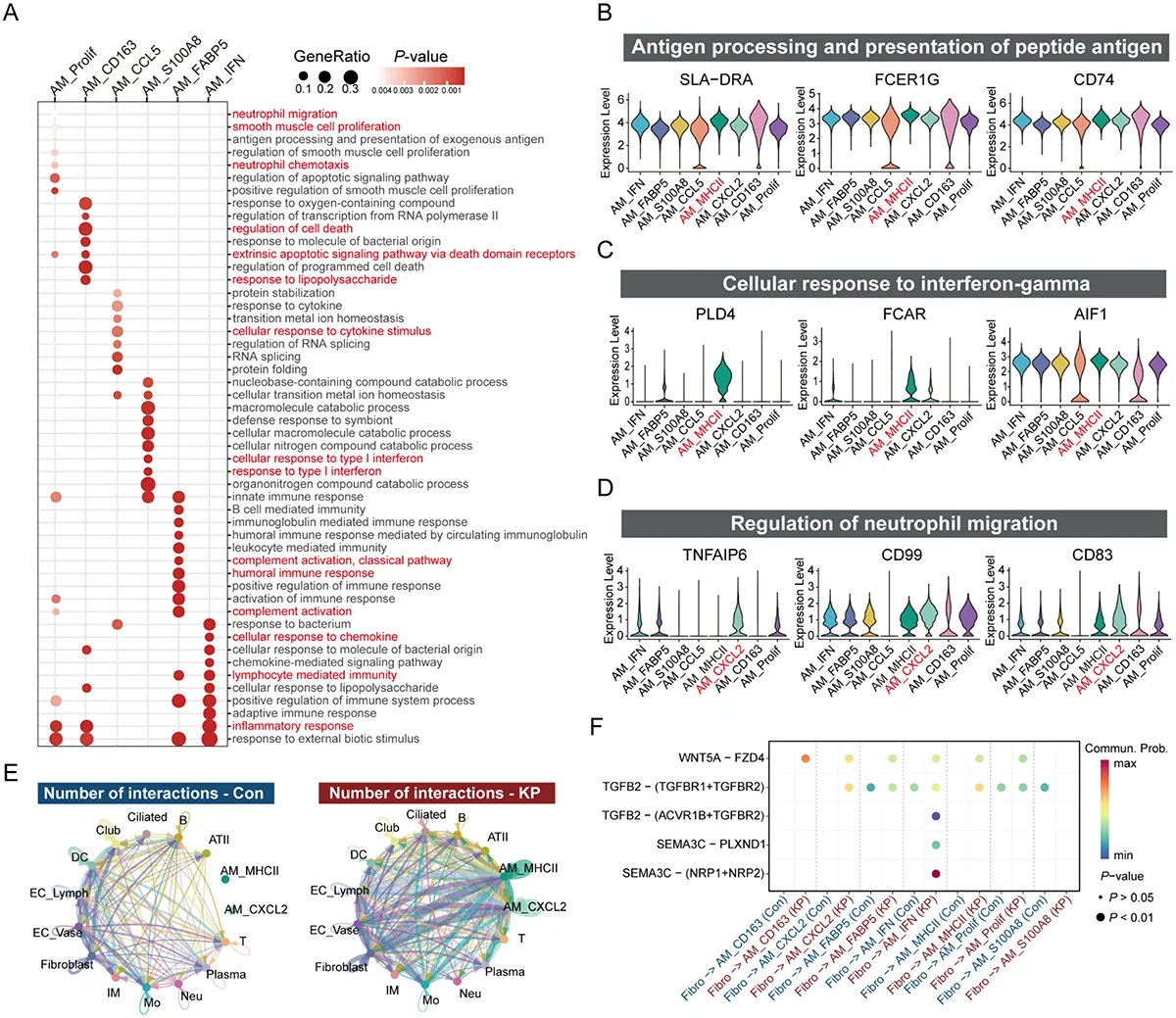
Functional characterization of am post-KP infection.

(A) Bubble plot of GO enrichment results for upregulated genes in the indicated AM subsets post-infection compared with controls. (B-D) Violin plots showing the expression distribution of immune pathway-related genes among AM subsets. (E) Network quantification of ligand-receptor interactions between AM and other cells, with thicker edges indicating stronger changes in interaction strength. (F) Bubble plot displaying the altered ligand-receptor pairs between fibroblasts and macrophages post-infection.

During infection, macrophage pool replenishment occurs not only through selective Mo conversion but also via local AM proliferation and differentiation [31]. To investigate the roles of two emergent subsets (AM_MHCII and AM_CXCL2), we analyzed their DEGs. AM_MHCII highly expressed antigen-processing/presentation-related genes (e.g. *SLA-DRA*, *FCGR1G*, and *CD74*), consistent with a potential antigen-presenting capacity (Figure 4(B); Table S5). This subset also upregulated IFN-γ-responsive genes (e.g. *PLD4*, *FCAR*, and *AIF1*). Given that *PLD4* is enriched in M1 macrophages [32], these results suggest that AM_MHCII may possess pro-inflammatory traits alongside antigen presentation (Figure 4(C)). AM_CXCL2 showed high expression of inflammatory mediators and genes implicated in neutrophil migration (e.g. *TNFAIP6*, *CD99*, and *CD83*), indicating a potential role in regulating neutrophil recruitment (Figure 4(D)).

Cell communication analysis revealed that newly emerged macrophage subsets (AM_MHCII and AM_CXCL2) exhibited enhanced interaction networks with other cell types, with their signaling patterns showing highly similar pathway types and interaction strengths (Figure 4(E), Figure S3D-F). These subsets established new or enhanced communication pathways, including those related to fibrosis and immunosuppression (TGF-β signaling), and pathways involved in repair (EGF), proliferation (APRIL), and chemotaxis (CCL; Figure S3D). Interactions between the two emerged AM and B cells / plasma cells may occur through upregulated TNF receptor-ligand pairs (TNFSF13B-TNFRSF13C/TNFRSF13B), which are known to play crucial roles in B cell biology (Figure S3E and F) [33]. In its interactions with most immune cells, the interaction between macrophage migration inhibitory factor (MIF) and its receptor complex CD74+CXCR4/CD44 was enhanced (Figure S3E and F). MIF is a proinflammatory cytokine implicated in immune cell recruitment [34]. The newly identified MDK-NCL pathway showed enhanced interactions with all cell types except Neu (Figure S3E and F). This pathway has been reported as a key mechanism in liver regeneration [35], suggesting its potential role in pulmonary infection immunity. Furthermore, B cells, Mo, and Neu enhanced C3-(ITGAM+ITGB2) complement-receptor interactions, which may mediate immune cell adhesion and migration during lung injury (Figure S3D-F) [36]. Additionally, KP infection enhanced cellular communication between fibroblasts and remodeled AM subsets through WNT5A, TGF-β, and SEMA3C pathways. Specifically, the ligand-receptor pairs WNT5A-FZD4 and TGFB2-(TGFBR1+TGFBR2), which are associated with fibroblast differentiation and proliferation, were significantly upregulated in the newly emerged AM subsets (Figure 4(F)). This suggests a shift toward a pro-fibrotic phenotype in macrophage-fibroblast crosstalk during KP infection.

In summary, KP infection is associated with extensive AM apoptosis and a severe disruption of macrophage homeostasis. The remodeled macrophage subsets display distinct transcriptional and cellular communication profiles, suggesting their potential antigen-presenting capacity, inflammatory mediation, and pro-fibrotic propensity.

### KP infection shapes the CD8^+^γδ T and CX3CR1^−^CD8αα^+^ T cells in a more activated status

The proportion of T cells among immune cells increased post-KP infection, with similar results obtained via immunohistochemistry (Figure 5(A)). To systematically characterize the changes in the T cell compartment, we identified 7 subsets based on the gene expression profiles: CD8^+^γδ T (*CD3E*^*+*^ *CD8A*^*+*^ *CD8B*^*+*^ *TRDC*^*+*^), CX3CR1^−^CD8αα^+^ T (*CD3E*^*+*^ *CD8A*^*+*^), CX3CR1^+^CD8αα^+^ T (*CD3E*^*+*^ *CD8A*^*+*^ *CX3CR1*^+^), CD4^+^ T (*CD3E*^*+*^ *CD4*^*+*^), Proliferating T (*CD3E*^*+*^ *TOP2A*^*+*^ *STMN1*^*+*^), CD8^−^γδ T (*CD3E*^*+*^ *CD8A*^−^ *CD8B*^−^ *TRDC*^*+*^), GZMB^+^CD8^+^ T (*CD3E*^*+*^ *CD8A*^*+*^ *CD8B*^*+*^ *GZMB*^*+*^; Figure 5(B,C); Table S4).

**Figure 5.**
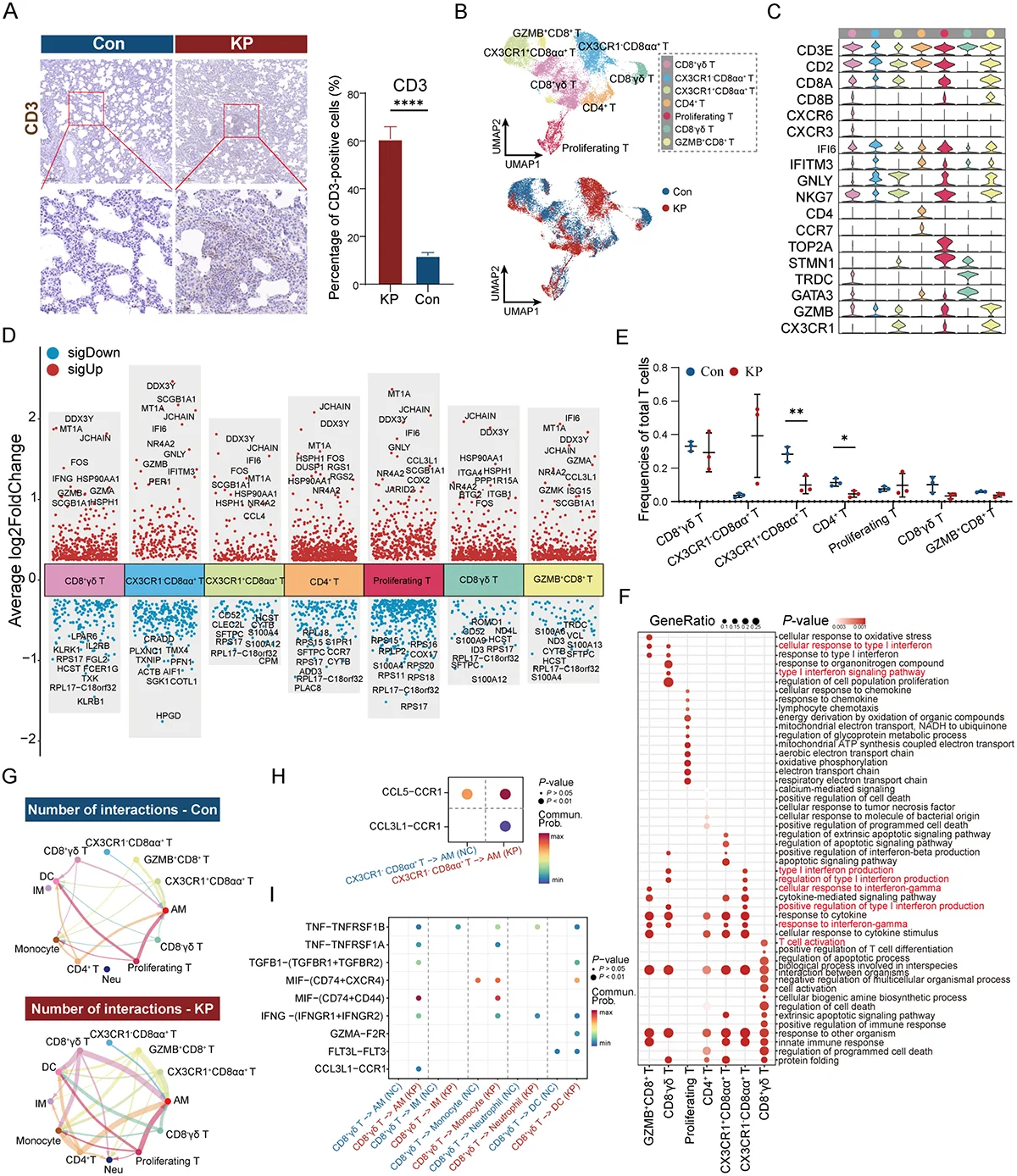
T cell responses to KP infection in porcine lungs.

(A) CD3 expression in lung tissues from Con and KP-infected groups. For quantification, eight random visual fields per sample were selected to calculate the percentage of CD3 positive cells. ****p < 0.0001. (B) UMAP visualization of T cell subsets and their cluster identities. (C) Violin plots showing marker gene expression across T cell subsets. (D) Top 10 differentially expressed genes in each T cell subset post-infection. (E) Abundance changes of T cell subsets post-KP challenge. The plot shows the percentage of each T cell subset among total T cells for individual biological replicates in the Con and KP groups (*n* = 3 per group). Horizontal lines indicate the group means. Statistical significance for the difference between the Con and KP groups for each subset was determined by the unpaired, two-tailed Wilcoxon rank-sum test. *p < 0.05, **p < 0.01. (F) Bubble plot of GO enrichment for upregulated genes in each T cell subset. (G) Network analysis of altered ligand-receptor interactions between T cell and myeloid cell populations. (H-I) Bubble plot of significant ligand-receptor pairs between CX3CR1-CD8αα+ T cells (H) CD8+γδ T cells (I) and myeloid cells.

Further enrichment analysis revealed that DEGs in T cell subsets post-infection were enriched in pathways related to immune responses with distinct effector functions (Figure 5). Our analysis identified two functionally distinct CD8αα^+^ T cell populations that exhibited divergent responses to KP infection. While CX3CR1^+^CD8αα^+^ T cells decreased post-infection and their DEGs were enriched in cytokine response and apoptosis pathways, CX3CR1^−^CD8αα^+^ T cells increased in abundance (Figure 5(E,F)). Compared to other phenotypic T-cell subsets, CX3CR1^−^CD8αα^+^ T cells exhibited high expression of *PER1*, transcription factors *STAT4* and *EOMES*, as well as the surface marker *IL18R1*, consistent with the currently defined Type 1 Cytotoxic T cell (Tc1) characteristics of CD8^+^ Tc subsets (Figure S4A). These molecules collectively promote IFN-γ production [37]. Enrichment analysis also indicated that this subset’s transcriptional profile was associated with cytotoxicity and interferon response pathways post-infection (Figure 5(F)). Cell-cell communication analysis indicated that CX3CR1^−^CD8αα^+^ T cells enhanced interactions with only AM and activated the ligand-receptor pair CCL5 (CCL3L1)-CCR1 post-infection, which implicates its roles in macrophage migration to inflammatory sites (Figure 5(G,H)), Figure S4B). Moreover, CX3CR1^−^CD8αα^+^ T cells exhibited high expression of activation markers (*SLA-DRA*, *CD69*, and *CD38*; Figure S4C). These results reveal that these T cells are in a highly activated state, indicating that the immune system is actively mobilizing T cells to combat KP infection.

Compared to humans and mice, swine possess high proportions of γδ T cells [38]. Similarly, γδ T cells accounted for the largest proportion of T cells in the lung under physiological state (Figure 5(E)). These cells were classified into CD8^+^γδ T and CD8^−^γδ T subsets (Figure 5(B)). Upon KP infection, the two γδ T subsets exhibited distinct functional profiles. The CD8^+^γδ T cell population showed a pronounced cytotoxic profile, characterized by elevated expression of granzyme B (*GZMB*), granulysin (*GNLY*), and the cytolytic regulator *NKG7*, suggesting enhanced killing capacity against infected cells (Figure 5(C)). Furthermore, cell-cell communication analysis revealed that CD8^+^γδ T cells exhibited stronger interactions with myeloid cells than CD8^−^γδ T cells post-infection (Figure 5(G)). Notably, the interactions between CD8^+^γδ T cells and myeloid cells primarily occur through three key signaling axes: (1) TNF-TNFRSF1B pathway, which can amplify inflammatory responses by enhancing mediator secretion [39]; (2) IFNG-(IFNGR1+IFNGR2) interaction, implicated in macrophage activation and neutrophil recruitment (Figure 5(G–I)) [40]; and (3) MIF-(CD74+CD44), which mainly mediates communication with monocytes/macrophages. In contrast, CD8^−^γδ T cells highly expressed IL-6 receptor (IL6R) that mediates Th17 differentiation, along with essential transcription factors GATA3 and MAF, and their DEGs post-infection were enriched in type I interferon signaling pathways (Figure 5(F), Figure S4D).

In summary, γδ T cells and CD8αα^+^ T cells constitute major T cell populations in the porcine lung under physiological homeostasis. Following KP infection, CX3CR1^−^CD8αα^+^ T cells transition to a highly activated state, while CD8^+^γδ T cells, which display a cytotoxic profile, exhibit enhanced cellular interactions with myeloid cells.

### KP promotes pulmonary fibrosis through ROS-mediated upregulation of glycolysis

Pulmonary epithelial cells are essential for maintaining lung homeostasis, contributing to barrier function, tissue repair, and mucosal immunity, while their dysregulation is implicated in various respiratory pathologies [41,42]. Single-cell analysis delineated four epithelial subsets: ATII, Fibroblast, Club, and Ciliated cells (Figure 6(A)). Notably, fibroblasts were the only stromal cell population that expanded post-infection, exhibiting marked upregulation of collagen family proteins (*COL1A1*, *COL1A2*, *COL3A1*) and proliferation markers (*TOP2A*, *CCND1*, *E2F1*) (Figure 6(B,C)) and Figure S4E). Histopathological evaluation via Sirius Red staining confirmed pronounced collagen deposition in infected lungs (Figure 6(D)). These findings suggest that KP infection stimulates proliferation and activation of lung fibroblasts, leading to excessive collagen synthesis and secretion, and ultimately pulmonary fibrosis.

**Figure 6.**
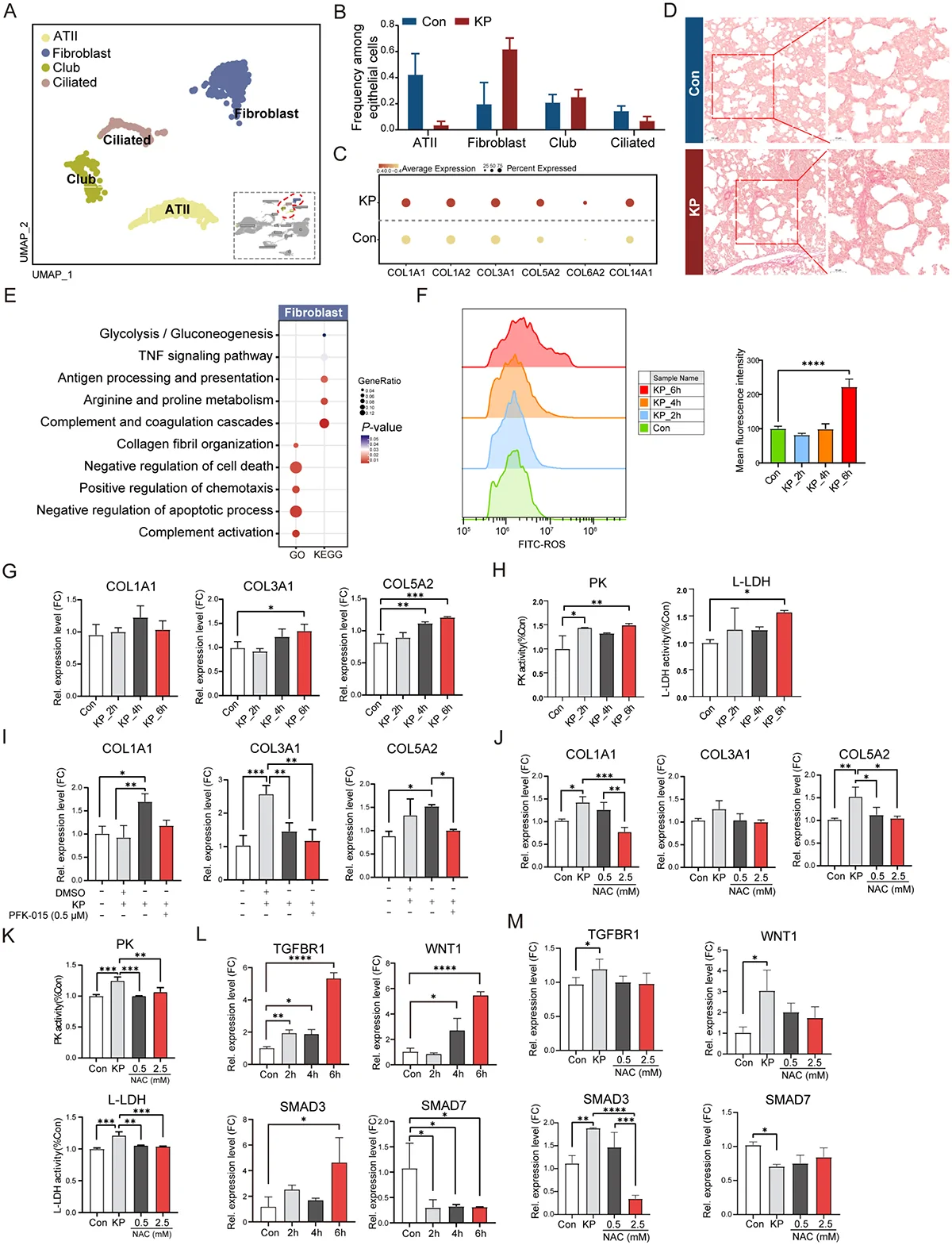
KP infection-induced pulmonary fibroblast remodeling.

(A) UMAP visualization of epithelial cell subsets with cluster annotations. (B) Proportional abundance of epithelial subsets post-infection. Bars show percentage among total epithelial cells (mean ± SEM, *n* = 3 per group). Significance between the Con and KP groups for each subset was determined by the unpaired, two-tailed Wilcoxon rank-sum test. *p < 0.05, **p < 0.01. (C) Bubble plot of collagen gene expression changes in fibroblasts. (D) Sirius Red staining showing collagen fiber distribution and deposition. (E) GO/KEGG enrichment of upregulated fibroblast genes post-infection. (F) Flow cytometric detection of KP infection-induced dynamic changes in ROS levels in porcine primary fibroblasts. Data are presented as mean fluorescence intensity (MFI) (mean ± SEM, *n* = 3). Statistical significance of each KP group vs the Con was determined by one-way ANOVA with Dunnett’s post hoc test. ****p < 0.0001. (G, I, J) RT-qPCR analysis of collagen gene expression. Data are fold change vs Control (mean ± SEM, *n* = 3). (H, K) PK and L-LDH activity assays in KP-infected fibroblasts (mean ± SEM, *n* = 3). (L, M) RT-qPCR analysis of TGF-β/WNT pathway components (mean ± SEM, *n* = 3). For (G, H, I, J, K, L, M): Significance vs Control at each time point was determined by one-way ANOVA with Dunnett’s test. *p < 0.05, **p < 0.01, ***p < 0.001, ****p < 0.0001.

To further investigate the mechanisms underlying KP-induced pulmonary fibrosis, we examined fibroblast DEGs post-infection and found their significant enrichment in arginine/proline metabolism, collagen fibril organization, and glycolysis/gluconeogenesis pathways-all critically associated with fibrogenesis (Figure 6(E)). Flow cytometric analysis revealed significantly elevated ROS production in primary porcine lung fibroblasts post-KP infection, along with increased collagen expression, lactate dehydrogenase (LDH), and pyruvate kinase (PK) levels (Figure 6(F–H)). Treatment with the glycolytic inhibitor PFK-15, which blocks key glycolytic enzymes, suppressed KP-induced collagen upregulation (Figure 6(I)). Similarly, NAC-mediated ROS inhibition attenuated both glycolytic activity and collagen expression (Figure 6(J,K)). Furthermore, we found that the mRNA expression levels of key regulators in the fibrotic process-TGF-β receptor (TGFBR1), downstream SMAD3, and Wingless/Int-1 (WNT1)-were significantly increased after infection, while the key negative regulator of the TGF-β signaling pathway, SMAD7, was markedly downregulated (Figure 6(L)). When ROS was inhibited using NAC, the expression levels of *TGFBR1*, *SMAD3* and *WNT* were downregulated (Figure 6(M)). These results indicate that KP infection activates ROS-mediated TGF-β/SMAD and WNT pathways and aerobic glycolysis, synergistically promoting fibroblast activation and proliferation, thereby exacerbating lung injury and fibrosis.

### KP infection reduces the proportion of ATII and AT0, and their differentiation into ATI

ATII cells are critical components of the distal lung epithelium, capable of mediating innate immune responses and serving as progenitor cells for ATI cells to facilitate alveolar epithelial repair and regeneration [43]. Single-cell RNA-seq and immunofluorescence analysis revealed that KP infection reduced ATII cell numbers, leading to alveolar collapse and severe lung injury (Figure 6(B), 7(A)). Club cells, which possess stem cell-like properties, maintain respiratory epithelial integrity and function by regulating their own proliferation or that of other epithelial cell types. DEG enrichment analysis further revealed upregulated pathways associated with epithelial cell proliferation after KP infection, suggesting their potential role in mitigating ATII cell loss (Figure 7(B)).

**Figure 7.**
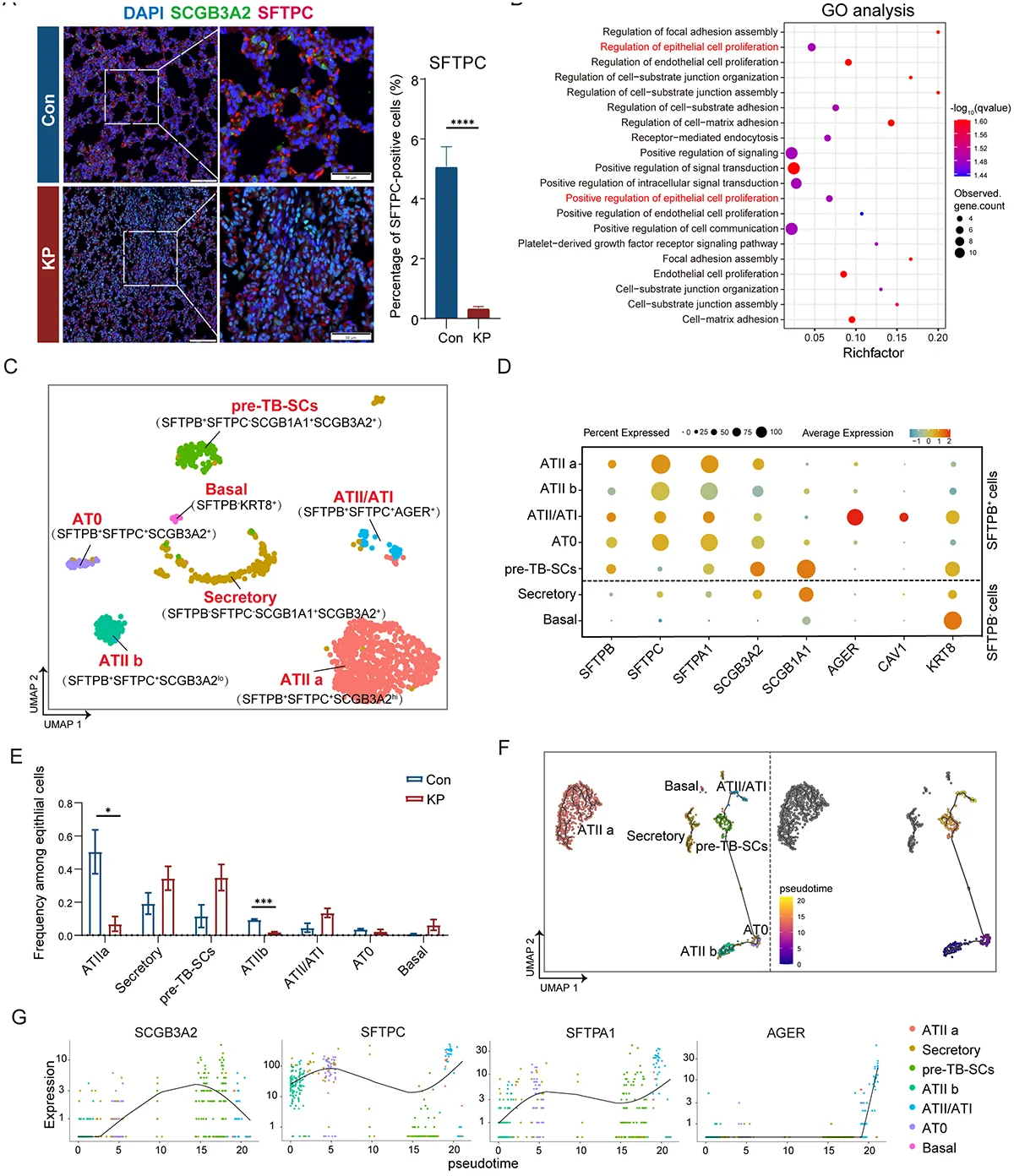
Characteristics of porcine distal airway epithelial cells and their changes post-infection.

(A) Expression of SCGB3A2 and SFTPC in the lungs of Con and KP-infected groups. For quantification, eight random visual fields per sample were selected to calculate the percentage of SFTPC positive cells. ****p < 0.0001. (B) Bubble plot showing the gene enrichment analysis of upregulated genes in Club cells after KP infection compared to the Con. (C) UMAP plot displaying the distribution and cluster information of epithelial cell subsets. (D) Bubble plot illustrating the scaled expression levels of epithelial cell type-specific genes. (E) Changes in the abundance of each epithelial cell population (mean ± SEM, *n* = 3). Significance between Con and KP groups for each subset was determined by the unpaired, two-tailed Wilcoxon rank-sum test. *p < 0.05, ***p < 0.001. (F) Pseudotime trajectory analysis of epithelial cell populations. (G) Changes in SCGB3A2, SFTPC, SFTPA1, and AGER along the pseudotime trajectory.

Anatomically, porcine and murine lung tissues exhibit significant structural divergence at the bronchial-alveolar junction [21]. To characterize porcine distal airway epithelial cell types and their potential differentiation during KP infection, we performed integrated dimensionality reduction and clustering analysis of Club and ATII cells (Figure 7(C)). Cell populations were annotated using established human distal airway epithelial classification markers [21], resulting in 7 distinct subpopulations: ATII a (*SFTPB*^*+*^ *SFTPC*^*+*^ *SCGB3A2*^*hi*^), ATII b (*SFTPB*^*+*^ *SFTPC*^*+*^ *SCGB3A2*^*lo*^), ATII/ATI (*SFTPB*^*+*^ *SFTPC*^*+*^ *AGER*^*+*^), AT0 (*SFTPB*^*+*^ *SFTPC*^*+*^ *SCGB3A2*^*+*^), pre-TB-SCs (*SFTPB*^*+*^ *SFTPC*^−^ *SCGB3A2*^*+*^ *SCGB1A1*^*+*^), Secretory (*SFTPB*^−^ *SFTPC*^−^ *SCGB1A1*^*+*^ *SCGB3A2*^*+*^), Basal (*SFTPB*^−^ *KRT8*^*+*^). Spatial mapping of *SFTPB* expression effectively demarcated proximal vs distal airway epithelial compartments in porcine lungs (Figure 7(D)), mirroring organizational patterns observed in human airways. Beyond the well-characterized classical epithelial cells ATII and ATI and distal airway cells (Secretory and Basal), we identified pre-terminal bronchiolar secretory cells (pre-TB-SCs and AT0), both recently discovered in human distal airways [44]. Following infection, the proportions of ATII and AT0 decreased while other cell populations increased (Figure 7(E)). The identification of these precursor cells in porcine lungs further demonstrates that the epithelial cell types in the distal airways of pigs are highly similar to humans, and can serve as a good model for studying distal airway diseases [21].

Pseudotime analysis revealed that ATII b cells with progenitor features exhibited differentiation potential toward AT0, accompanied by progressively increased expression of AT0 markers *SFTPC* and *SCGB3A2* (Figure 7(F,G)). The transitional AT0 cell population further differentiated toward an ATII/ATI phenotype, with monotonically increasing *AGER* expression along the pseudotime trajectory (Figure 7(F,G)). Following KP infection, both ATII b and transitional AT0 populations decreased, suggesting KP infection may disrupt the ATII-AT0-ATI differentiation program, causing developmental arrest or cell death and impairing proper ATI conversion (Figure 7(E,F)). These findings suggest that injury signals triggered by KP infection may suppress ATII progenitor differentiation into terminal cell states, impairing timely replenishment of severely depleted ATII/ATI populations and consequently exacerbating pulmonary epithelial barrier damage.

## Discussion

KP, especially hypervirulent and multidrug-resistant strains, causes severe infections in the respiratory, urinary, and circulatory systems in humans and important animal species [45,46]. Current knowledge regarding KP-induced immune responses predominantly comes from rodent studies (mainly using mouse models) [47]. Nevertheless, a critical limitation of these models is that the mouse respiratory system lacks respiratory bronchioles – a structural component that is a frequent site of pulmonary damage in humans. To address this gap and gain more clinically and physiologically relevant insights, we employed scRNA-seq to delineate functional and abundance changes in immune and structural cells in porcine lungs during KP infection, thereby establishing a comprehensive atlas of pulmonary cellular response induced by KP.

KP colonization of host organs depends on interactions between phagocytes (e.g. macrophages, Neu, and Mo) and bacteria, which collectively mediate pathogen clearance. Innate immunity is well recognized as a central mediator of host defense against KP: following infection, myeloid cells (Neu, Mo, and IM) rapidly activate and recruit to inflammatory foci. Consistent with human KP infection, this porcine model exhibits prominent neutrophilic infiltration [48]. Beyond confirming known pathways like TLR upregulation [49,50], our analysis revealed a potentially novel immune evasion strategy, in which KP infection downregulated the complement component C1Q in neutrophils. Given the established role of C1Q in opsonization and complement-mediated clearance of pathogens like *Streptococcus pneumoniae* [51], suppression in the porcine model suggests a targeted mechanism by which KP may impair complement-mediated clearance. This finding extends beyond the known KP strategies of resisting phagocytosis or NETosis [52] and highlights a previously underappreciated aspect of KP-neutrophil interaction that warrants further investigation.

IM, though less studied in infection, emerged as a dominant responding subset in our porcine model, mirroring observations in mice [9]. Here, we found that KP infection triggers IM recruitment in porcine lungs, with IFN signaling activation being a hallmark feature of IM post-KP infection. Mounting evidence reveals a complex interplay between KP pathogenesis and IFN signaling pathways, influencing both bacterial virulence and antimicrobial resistance. Type I IFN drives reciprocal activation of natural killer (NK) cells and macrophages: macrophages promote IFN-γ secretion by NK cells, which in turn enhances macrophage phagocytic and bactericidal activity against KP [50]. In adaptive immunity, type I IFN signaling facilitates naive T cell proliferation and activation, modulates T cell subset differentiation, and enhances B cell antigen recognition and uptake capacity against KP, thereby promoting antibody secretion [53]. Our finding that porcine lung IMs serve as a major source of IFN-I post-infection highlights their previously underappreciated role in infection biology. This function appears distinct from that of AM, thus warranting further mechanistic investigations in both porcine and murine models.

Under homeostasis, AM are pivotal for maintaining pulmonary immune quiescence and tissue integrity [28]. Despite the widely cited M1/M2 macrophage polarization paradigm, many studies report difficulties in distinguishing them at single-cell resolution. We annotated eight functionally distinct AM subsets in porcine lungs based on differential gene profiles. Initially, infection induced extensive apoptosis in multiple resident AM subsets (AM_CCL5, AM_CD163, AM_IFN, AM_Prolif), a shared pathogenic tactic observed in other pneumonias to disrupt the frontline defense [54–56]. Subsequent repopulation, as inferred from pseudotime analysis, involved both monocyte-derived AMs (AM_CCL5, AM_CD163) and the emergence of two novel, infection-specific subsets (AM_MHCII, AM_CXCL2). These new subsets displayed a hybrid phenotype, characterized by enhanced antigen presentation (MHCII), pro-inflammatory activity (PLD4), and chemotactic signaling (CXCL2), and established stronger interactions with adaptive immune cells. This remodeled AM pool, therefore, may represent a population that has undergone functional repolarization, shifting from a homeostatic surveillance state to an acutely activated phenotype geared toward enhanced pathogen clearance and immunomodulation. This acute phenotypic shift shares features with the altered macrophage states observed following other pulmonary infections [20,57].

While the innate immune response to KP infection has been extensively characterized [58], research on KP-induced cell-mediated immunity remains relatively limited, with most studies confined to mouse models. However, emerging evidence underscores the equally critical role of adaptive immunity in host defense against KP infection. Notably, mouse studies have established that T cells are indispensable for protective immunity against classical KP strains [59]. Consistent with reports of CD8αα^+^ T cell proliferation in human viral infections (e.g. HIV-1, hepatitis B) [60,61], we observed expanded CD8αα^+^ T cell populations post-KP infection in pigs, which are competent for cytotoxicity. Pigs possess higher γδ T cell numbers compared to other species [38], warranting in-depth investigation into their specific roles in combating KP infection. We identified CD8^+^γδ T cells as a relatively abundant subset in porcine lungs. CD8^+^γδ T cells mediate NK-like cytotoxicity and robust IFN-γ production in human pathologies (e.g. inflammatory bowel disease, tuberculosis, melanoma) [62,63]. During KP infection, CD8^+^γδ T cells demonstrate enhanced cytotoxic functionality and IFN-γ production capacity, along with intensified interactions with myeloid cells. This functional profile aligns with recent thymic studies showing neonatal γδ T cells outperform their αβ T counterparts in IFN-γ production [64]. We speculate that CD8^+^γδ T cells may play significant roles in combating bacterial infections, and future studies should discriminate CD8 expression in γδ T cell populations. These discoveries hold important implications for understanding adaptive immune regulation during pulmonary KP infection.

While existing studies of KP-induced immune responses in structural cells have predominantly focused on ATII cells, these investigations have been largely limited to cell line models [65], which do not replicate the *in vivo* microenvironment. Anatomical differences between mouse and human lungs further limit functional studies of respiratory bronchioles [66]. In our porcine KP infection model, we found that KP infection not only causes massive loss of ATII cells, leading to alveolar collapse and impaired repair, but also induces pulmonary fibrosis, resulting in irreversible lung damage. Notably, these pathological manifestations are consistent with hallmark features observed in human KP pneumonia patients [67,68]. Studies have shown that ROS levels are significantly elevated in pulmonary fibrosis, and ROS inhibition can alleviate fibrotic progression [69]. Consistent with this finding, we found KP infection upregulated fibroblast ROS production, key glycolytic kinases, and multiple collagen proteins, indicating a fibrotic trajectory in infected lungs. Inhibiting ROS reduced aerobic glycolysis, collagen production, and expression of TGF-β pathway-related molecules. TGF-β promotes pulmonary fibrosis by regulating myofibroblast differentiation/proliferation and fibrosis-related gene transcription via SMAD signaling [70]. In addition, we found that LDH and PK levels increased after KP infection, which is consistent with results in patients with bacterial pneumonia and other human cell lines infected with KP [71].

High-resolution sequencing technologies have revolutionized our understanding of distal airway architecture, uncovering novel structural cell types with critical roles in pulmonary homeostasis and repair. Recent studies on human distal airways have identified respiratory airway secretory (RAS) and AT0 cell populations that serve as progenitor cells and intermediate transitional states during both homeostasis and disease, revealing the complexity and significance of distal epithelial regenerative programs [21,66]. Consistent with these studies in humans, we also identified the pre-TB-SCs and AT0 cells in the distal airway in porcine lungs. However, the reduction of AT0 proportion post-infection may reflect KP-induced blockade of ATII-to-ATI differentiation, which warrants further investigation. In addition, contrary to the classical binary view of alveolar epithelial differentiation (ATII-ATI), we identified a subset of terminally differentiated cells exhibiting a hybrid *SFTPC*^*+*^ *AGER*^*+*^ phenotype. This challenges the prevailing paradigm that *SFTPC* expression is exclusively silenced upon ATI maturation [72]. This hybrid phenotype has also been reported in humans, revealing unexpected plasticity in terminally differentiated alveolar epithelium and suggesting unrecognized intermediate differentiation pathways [66]. These data confirm that pigs share anatomical and cellular similarity with humans, supporting their utility as models for respiratory disease and therapeutic testing. Meanwhile, these findings significantly advance our understanding of host immune responses to KP infection in distal airways.

This study is subject to several noteworthy limitations. Firstly, the limited sample size (*n* = 3 per group) and the use of pigs from a single age and sex cohort constrain the statistical power of the study. Future studies employing larger cohorts with diverse age and sex distributions will be required to improve the generalizability of the findings. Second, the analysis was confined to a single time point (96 h post-infection), which limits a comprehensive understanding of the dynamic trajectory of the host response. Third, the cell communication analysis relied on the CellChatDB. human database to infer ligand-receptor interactions for porcine cells, a methodology consistent with current practices in the field [24]. However, potential interspecies specificity differences might impact the precision of these inferences. To achieve a more comprehensive and accurate elucidation of the infection mechanisms, subsequent investigations should incorporate longitudinal designs with multiple time points, employ larger sample sizes, and utilize or develop analytical tools specifically validated for cross-species applications. Fourth, while direct effects of KP on macrophage apoptosis and pro-fibrotic effects on fibroblasts were verified in our in vitro model, we still cannot fully distinguish whether these effects are direct or indirect. Further investigations are warranted to dissect the underlying mechanisms in the intact lung microenvironment. Furthermore, the cell-cell communication patterns identified via CellChat remain computational predictions without experimental verification, the specific regulatory axes therefore warrant further functional validation.

## Conclusion

Using scRNA-seq, we established a transcriptional atlas of porcine lungs infected with high-virulence and multidrug-resistant KP, which reveals extensive AM apoptosis associated with KP infection. The remodeled AM pool exhibited enhanced immune functionality and cellular crosstalk, while CD8^+^γδ T cells strengthened interactions with myeloid cells post-infection. Post-infection lung fibroblasts showed elevated reactive ROS and activated aerobic glycolysis, with coordinated TGF-β/SMAD and WNT signaling linked to pulmonary fibrogenesis. Concurrently, reduced ATII cells and transitional AT0 populations exacerbated KP-induced impairment of the lung epithelial barrier. These findings delineate the interplay between immune and structural lineages during KP infection. By leveraging a large mammalian model, we uncovered key mechanisms of KP pathogenesis, overcoming limitations of rodent models and advancing understanding of KP-host interactions. Thus, our study provides a potential translational framework for developing interventions against high-virulence and multidrug-resistant KP.

## Supplementary Material

Clean_Supporting Information.docx

## Data Availability

The scRNA-seq data generated in this study have been deposited in the Genome Sequence Archive (GSA) under the accession number CRA039450 and are publicly accessible at https://ngdc.cncb.ac.cn/gsa/browse/CRA039450. The custom analysis scripts used in this study are archived on Zenodo under a CC-BY open license. The code is publicly available at https://doi.org/10.5281/zenodo.20192192 (DOI: 10.5281/zenodo.20192192). The source code is also publicly available in the GitHub repository at https://github.com/ww1186372087-lab/Kp-Infection-Porcine-Lung-scRNA. The supplementary materials and raw data associated with this study can be accessed on https://doi.org/10.6084/m9.figshare.32298105.v3 [73].
