# Supplementary material for: Single-cell transcriptomic atlas uncovers immune/structural lineage remodeling and fibrosis in Klebsiella pneumoniae-infected porcine lungs
Source: Virulence. 2026 Oct 12;17(1):2747487. doi: 10.1080/21505594.2026.2747487 (PMC13682406; doi:10.1080/21505594.2026.2747487)
Supplement: Supplementary file 1 [file KVIR_A_2747487_SM3776.docx]

Supporting materials

**Table S1** The *WZI* allele sequencing comparison of bacterial strains.

**Table S2** Primers for the identification of KP.

**Table S3** Drug sensitivity test results.

**Table S4** Marker genes used for the identification and annotation of all major cell clusters reported in this study.

**Table S5** Differentially Expressed Genes used for the identification and annotation of all cell clusters reported in this study.

**Table S6** The primer sequence for RT-qPCR.

Table S1 The *WZI* allele sequencing comparison of bacterial strains.

| Locus | Allele | Length | Contig | Start position | End position | Attributes |
| --- | --- | --- | --- | --- | --- | --- |
| *WZI* (*WZI* outer) membrane) proteir of cps cluster) | 72 | 447 | Query | 62 | 508 | K-type: K2 |

Table S2 Primers for the identification of KP.

| Gene | Forward (5′-3′) | Reverse (5′-3′) |
| --- | --- | --- |
| aerobactin | GCATAGGCGGATACGAACAT | CACAGGGCAATTGCTTACCT |
| iroB | ATCTCATCATCTACCCTCCGCTC | GGTTCGCCGTCGTTTTCAA |
| rmpA2 | GTGCAATAAGGATGTTACATTA | GGATGCCCTCCTCCTG |
| Peg-344 | CTTGAAACTATCCCTCCAGTC | CCAGCGAAAGAATAACCCC |
| rmpA | GCATAGGCGGATACGAACAT | CTTGCATGAGCCATCTTTCA |
| iucA | AATCAATGGCTATTCCCGCTG | CGCTTCACTTCTTTCACTGACAGG |
| O1 | GATTTCACTTTCCGGGCAAC | GGCTTGCTGAATCACAAGAC |
| O2ac | AAACATCGCTGACTCGAGTC | CGACTATGATCGTACCAACG |
| O1/O2-A | CGCTATAAGAGCAGCATGCTAG | CGATATCACCTACTGCCAGA |
| O1/O2-B | TTGTTGAGCCTGACAGGATC | GCCATTGCTTGCTTGTACAG |

Table S3 Drug sensitivity test results. R: Resistant; I: Intermediate; S: Sensitive

| Antibiotic | Abbreviation | Sensitivity |
| --- | --- | --- |
| Cephalothin | CN | I |
| Ceftazidime | CAZ | S |
| Ampicillin | AMP | R |
| Penicillin | PEN | R |
| Oxacillin | OX | R |
| Lincomycin | MY | R |
| Polymyxin B | PB | I |
| Norfloxacin | NOR | I |
| Ciprofloxacin | CIP | S |
| Vancomycin | VAN | R |
| Trimethoprim-sulfamethoxazole | SXT | R |
| Erythromycin | E | R |
| Azithromycin | AZI | I |
| Minocycline | MI | S |
| Streptomycin | S | I |
| Kanamycin | KAN | S |
| Tetracycline | TET | S |
| Cefuroxime sodium | CXM | S |
| Cefoperazone | CPZ | S |
| Gentamicin | GEN | I |
| Amikacin | AMK | S |
| Ceftriaxone | CTR | S |
| Cefazolin | CZ | I |

Table S6 The primer sequence for RT-qPCR.

| Genes | Primers | Sequences (5’ to 3’) |
| --- | --- | --- |
| Actin | Forward | GAGAAGAGCTACGAGCTGCC |
|  | Reverse | CGTAGAGGTCCTTGCGGATG |
| COL1A1 | Forward | TGAAGCTGGTCCCCAAGGA |
|  | Reverse | AATACCAGGAGCGCCGTTG |
| COL3A1 | Forward | CTAGCCGAGCTTCCCAGAAC |
|  | Reverse | TCCCCAGTGTGTTTAGTGCAA |
| COL5A2 | Forward | CCCAGCAGTCAGCCTCTTTG |
|  | Reverse | GCATGTGAACAGTGATGGCA |
| TGFBR1 | Forward | ACCTAATTCCGCGAGACAGG |
|  | Reverse | AAGGCCAGATGGTGGCTTTC |
| WNT1 | Forward | TACTGCACGAGTGCCTGTGA |
|  | Reverse | TTATGTGCCAGCGAACCTGT |
| SMAD3 | Forward | CGAGAGTTGAGGCGAAGTTTG |
|  | Reverse | AAGGGCAGGATGGACGACAT |
| SMAD7 | Forward | CAACCCCCATCACCTTAGCC |
|  | Reverse | GCATCTGGACAGTCAGTTGGT |
